# Editorial: Novel insights into the modulation of protein function by lipids and membrane organization

**DOI:** 10.3389/fcell.2025.1607512

**Published:** 2025-04-24

**Authors:** Florina Zakany, Zsolt Török, Tamas Kovacs

**Affiliations:** ^1^ Department of Biophysics and Cell Biology, Faculty of Medicine, University of Debrecen, Debrecen, Hungary; ^2^ Laboratory of Molecular Stress Biology, Institute of Biochemistry, HUN-REN Biological Research Centre, Szeged, Hungary

**Keywords:** direct lipid-protein interactions, indirect lipid-protein interactions, membrane organization, lipid rafts, cholesterol, ceramide, fine-tuning, ion channels

Although the plasma membrane was originally thought to solely represent a passive diffusion barrier separating the intracellular and extracellular spaces, a growing body of evidence supports the active contribution of lipids and membrane organization to regulating the structure and function of transmembrane proteins. Membrane lipids can exert such actions through direct effects, that is, ligand-like binding of lipids to specific binding sites on proteins, and indirect mechanisms mediated by alterations in membrane biophysical properties (fluidity, hydration, lipid order, thickness, elasticity, lateral pressure and dipole potential). Furthermore, the tendency of biological membranes to segregate laterally into dynamic nano- and microdomains such as cholesterol-enriched lipid rafts and ceramide platforms, and their changes in response to altered lipid composition, add a further level of complexity to the active modulatory role of lipids in the functional regulation of proteins. By exerting permissive and cooperative actions on conformational changes associated with the activation of transmembrane proteins, such direct and indirect protein-lipid interactions modify a wide variety of cellular functions such as signaling pathways, apoptosis, cell adhesion and migration, synaptic transmission, cytoskeletal organization, protein sorting, pathogen entry, formation of amyloid plaques or extracellular vesicles, stress and immune responses (Zakany et al., 2020; Levental and Lyman, 2023). This Research Topic includes four studies that provide novel approaches and ideas about the intrinsic connection between functional modulation of proteins and lipids and the organization of cellular membranes.

Membrane bilayers have recently been proposed to act as extraordinarily precise, “finely-tuned molecular machines” due to the compositional complexity of the lipid species found in membranes and their numerous specific interactions with membrane proteins (Dingjan and Futerman, 2021). Recent advances in high-resolution X-ray crystallography and cryo-EM, along with molecular dynamics simulations, have led to the description of a large number of such modulatory direct lipid-protein interactions (Corradi et al., 2019; Zakany et al., 2020). The perspective of Dingjan and Futerman proposed that the ceramide-sensing negative feedback mechanism to avoid toxic ceramide accumulation in the endoplasmic reticulum is a manifestation of such a “finely-tuned” system through direct interactions between serine palmitoyl transferase with the inhibitory ORMDL subunit (SPT-ORMDL) and ceramide. After summarizing related literature cryo-EM data, the authors introduced a simplified computational model of the endoplasmic reticulum-localized sphingolipid flux, and analyzed the energetic contribution of single residues to ceramide binding by calculating the docking score and the predicted binding free energy for mutant SPT-ORMDL complexes, which, while not being validated experimentally in the study, are in agreement with recently published experimental data. Furthermore, the authors placed their findings in the context of intriguing mechanistic questions about the evolution of the (glyco)sphingolipid biosynthetic pathway (Biran et al., 2024).

The nicotinic acetylcholine receptor (nAchR), the paradigm member of the pentameric ligand-gated ion channel superfamily of neurotransmitter receptors, is also modulated by membrane lipids and, in turn, exerts influence on its membrane surroundings. The neuromuscular synapse, which contains a large quantity of nAChR molecules in an exceptionally high density, represents a unique case that is distinct from the conventional raft-versus-non-raft organization but rather constitutes a large two-dimensional nAChR ‘picket’ with gap-filling lipids under the influence of the receptors, which develops during neurodevelopment and synaptogenesis. The review by Barrantes reviewed the current knowledge on the mechanisms of nAChR modulation by membrane lipids both through direct and indirect mechanisms and pointed to distinctive singular modes of crosstalk with the membrane milieu resulting from its large abundance and density at the neuromuscular synapse, and characterized by an organization of numerous directly bound ‘non-annular’ and more dynamic ‘annular’ lipids complementing recent reviews in the field (Borroni and Barrantes, 2021; Barrantes, 2023) from a biophysical perspective. Although many lipid interaction sites have been suggested by biochemical-biophysical and atomistic structural studies of the nAChR, their pathophysiological relevance has not yet been demonstrated.

Mechanosensitive ion channels, which play a substantial role in endothelial mechanotransduction and thus blood pressure regulation, are activated by mechanical forces, such as shear stress, a frictional force generated by the blood flow and membrane tension generated by stretch (Beverley et al., 2025) and their endothelial stiffening-induced functional alterations may contribute to cardiovascular disease and aging (Aguilar et al., 2022). The comprehensive review of Beverley and Levitan summarized how cholesterol, which is intrinsically related to the pathogenesis of cardiovascular disease, modifies the functions of mechanosensitive ion channels including i) Piezo channels by altering their microenvironment and regulating their interaction with auxiliary proteins such as stomatin-like protein 3 (STOML3); ii) various Kir channels by direct binding at different sites; iii) TRPV4 by direct binding and partitioning into caveolar microdomains; and iv) voltage-regulated anion channels (VRAC) by membrane biophysics-related mechanisms. While the authors convincingly demonstrated the relevance of cholesterol-mediated modulation of mechanosensitive ion channels based on computational and simple cellular studies, this needs to be supported in the future by *in vivo* studies, which are only sporadic in animal models and almost completely missing in humans.

Cyclodextrins are cyclic oligosaccharides capable of forming soluble complexes with hydrophobic compounds such as lipids or drugs and are thus frequently used as carriers in pharmaceutical formulations (Kali et al., 2024). Recently, however, these compounds have been acknowledged as potential active therapeutic compounds (Kovacs et al., 2022a). Consistently, methyl-β-cyclodextrin, the most effective cyclodextrin to complex cholesterol, was recently shown both *in vitro* and *in vivo* to exert analgesic effects via inhibition of TRPV1 and TRPA1 channels through membrane cholesterol depletion and lipid raft disruption (Horvath et al., 2020). As a continuation of this study, Nehr-Majoros et al. showed that other cyclodextrins, without the hemolytic and toxic effects of methyl-β-cyclodextrin and thus more suitable for human application, are also able to reduce TRPV1 and TRPA1 activation by altering the membrane microenvironment through cholesterol depletion in simple cellular models. This study is consistent with i) recent reports describing alternative means of raft disruption and consequent antinociceptive effects (Payrits et al., 2024); and findings that cyclodextrin derivatives with relatively low *in vitro* cholesterol-complexing ability can affect ion channel activity (Kovacs et al., 2021) and exert beneficial actions in diseases as previously suggested in SARS-CoV-2 infection (Kovacs et al., 2023). While this study introduced non-cytotoxic cyclodextrins as promising plausible candidates for human analgesia, their efficacy should be verified in future *in vivo* and, subsequently, in human studies. Furthermore, potential off-target and side effects should be investigated.

Taken together, the manuscripts in this Research Topic highlight the extensive and diverse nature of biologically relevant lipid-protein interactions. However, this field of research still faces numerous major challenges and open questions that include those listed below. 1) Although computational and atomistic structural tools have identified innumerable potential direct lipid-protein interactions, these should be verified in more realistic membranes in MD simulations, with optimized sample preparation protocols for cryo-EM, and, particularly, experimentally (Muller et al., 2019; Biou, 2023). 2) The examination of the biophysical properties of membranes and their biological relevance currently represents a rather neglected area of research, which may be related to the generally unmet need for measurement techniques suitable for high-throughput and subcellular studies in living cells such as recently described (Danylchuk et al., 2021; Zakany et al., 2021; Andronico et al., 2024; Wong and Budin, 2024; Szabo et al., 2025). 3) The lateral organization of membranes still represents an unsolved mystery, particularly in intracellular membranes (Levental et al., 2020; Wang et al., 2020). 4) Most importantly, the contribution of membrane lipid-related alterations, although widely recognized, is very far from being understood in the pathogenesis of diseases that are intrinsically related to alterations in membrane lipid composition such as tumors, metabolic, neurodegenerative or lysosomal storage disorders. This is further aggravated by the paucity of animal and human studies in the field due to the limited translatability of biophysical insights gained with *in vitro* or simple cellular models. Nevertheless, a continuously growing number of studies, including the four published in this Research Topic, support the hypothesis of the therapeutic applicability of membrane lipid therapy, i.e., approaches that target the abnormal lipid homeostasis and modify the membrane lipid composition, structure, and lateral nanodomain organization of the cell membrane in human pathological conditions (Casares et al., 2019; Kovacs et al., 2022b).
